# Sanctuary policies and type 2 diabetes medication prescription trends among community health center patients

**DOI:** 10.1093/haschl/qxae178

**Published:** 2025-01-21

**Authors:** Salome Goglichidze, Wanjiang Wang, Louisa H Smith, David Ezekiel-Herrera, John D Heintzman, Miguel Marino, Jennifer A Lucas, Danielle M Crookes

**Affiliations:** Department of Public Health and Health Sciences, Bouvé College of Health Sciences, Northeastern University, Boston, MA 02115, United States; Department of Public Health and Health Sciences, Bouvé College of Health Sciences, Northeastern University, Boston, MA 02115, United States; Department of Public Health and Health Sciences, Bouvé College of Health Sciences, Northeastern University, Boston, MA 02115, United States; Roux Institute, Northeastern University, Portland, ME 04101, United States; Department of Family Medicine, Oregon Health & Sciences University, Portland, OR 97239, United States; Department of Family Medicine, Oregon Health & Sciences University, Portland, OR 97239, United States; Department of Family Medicine, Oregon Health & Sciences University, Portland, OR 97239, United States; Department of Family Medicine, Oregon Health & Sciences University, Portland, OR 97239, United States; Department of Public Health and Health Sciences, Bouvé College of Health Sciences, Northeastern University, Boston, MA 02115, United States; Department of Sociology and Anthropology, College of Social Sciences and Humanities, Northeastern University, Boston, MA 02115, United States

**Keywords:** type 2 diabetes, diabetes medications, prescriptions, sanctuary policies, immigrant health, access to prescriptions, community health centers

## Abstract

Immigrants in the United States are at increased risk of diabetes-related complications due to delayed diagnoses compared with US-born individuals. Immigration-related federal policies may support immigration enforcement activities and restrict some immigrants' access to health insurance and other publicly funded resources. Conversely, state and county-level sanctuary policies may reduce the fear of deportation and increase mobility in the community, improving the accessibility of essential pharmacological treatment for type 2 diabetes patients. This retrospective cohort study estimated the odds of receiving glucose-lowering medication prescriptions by the county's sanctuary policy environment for patients within a nationwide network of community health centers. We did not find statistically significant associations between sanctuary policies and annual prescription rates. The associations were not modified by nativity or race/ethnicity. Notably, compared to US-born patients, immigrants had higher odds of receiving prescriptions regardless of the sanctuary policy environment, emphasizing other potential influences on the receipt of anti-diabetes prescriptions for community health center patients.

## Introduction

Type 2 diabetes is a burden on health systems due to the increasing prevalence, duration, and cost of the illness.^1,2^ In 2021, of the 38 million people with diabetes in the United States, 90%-95% had type 2.^3^ Since 2002, the incidence of type 2 diabetes has increased significantly in the United States, with American Indian/Alaska Native, Latine, non-Latine Black, and non-Latine Asian individuals having higher rates of type 2 diabetes compared to non-Latine Whites in 2019-2021.^4,5^ Along with diet and physical activity, pharmacological treatment with insulin or oral hypoglycemic agents (OHAs) is a crucial part of type 2 diabetes care.^6-11^

In addition to other social determinants, nativity and documentation status can significantly influence diabetes awareness and receipt of appropriate type 2 diabetes care. Compared to US-born individuals, immigrants have higher odds of undiagnosed diabetes, decreased likelihood of treatment with insulin, and are at increased risk for diabetes complications, even after accounting for health insurance status.^12,13^ Documentation status may further impact healthcare access. For example, undocumented immigrants from Mexico are less likely to have usual sources of care than documented immigrants from Mexico.^14^ In qualitative research, undocumented African women have cited their documentation status as a deterrent to seeking formal healthcare.^15^ Understanding the factors contributing to differences in diabetes burden and treatment between immigrant and US-born populations is necessary to reduce the existing gaps.

Studies on healthcare access, non-specific to diabetes, have documented that policies and practices toward immigrants affect their ability to receive care and treatment.^16-21^ Immigrant-related health policies at federal and state levels influence immigrants' access to health insurance and healthcare (eg, Medicaid).^16,17^ Furthermore, immigration enforcement programs, such as the Secure Communities program and the 287(g) program, increase immigration enforcement at the community level and may result in lower care-seeking among undocumented immigrants or immigrants living in mixed-status families (ie, families with members of different citizenship or immigration statuses).^18-21^ For example, both programs have had negative physical and mental health impacts on Latine immigrants.^16,18^ Additionally, increased US Immigration and Customs Enforcement (ICE) activities within a state have been associated with a decreased likelihood of having a regular care provider or annual checkups for Latine adults (including immigrants and non-immigrants) but not for non-Latine US residents, reflecting the spillover effect of immigration-related policies on non-immigrant Latines.^21^ Fear about immigration enforcement and the effects of this fear on healthcare access have been documented among immigrants of different race/ethnicities and from different places of origin.^22-24^

Conversely, sub-federal level sanctuary policies can enhance immigrants' well-being.^25-27^ Sanctuary policies, aimed at protecting immigrants and refugees, can range from signaling that a locale is welcoming to immigrants to explicitly limiting local law enforcement's cooperation with ICE.^27^ For example, county-level sanctuary policies can restrict the routine reporting of the documentation status of people who encounter police, thus reducing fears of deportation, increasing mobility within the community, and potentially increasing willingness to seek healthcare.^28-30^ In a study of children with immigrant families, living in states with sanctuary policies was associated with a decreased likelihood of having unmet medical needs.^25^ In another study of Mexican immigrants living in two sanctuary areas, undocumented immigrants with diabetes had achieved similar clinical outcomes and reported similar healthcare experiences as documented immigrants and US-born Mexican Americans.^29^

There is a limited body of research on sanctuary policies (and immigrant-related policies more broadly) and diabetes-related outcomes.^27,31^ Most studies have instead focused on the effects of nativity and documentation status on access to health services and different health outcomes, especially among Latine immigrants.^16,32-36^ As a medically vulnerable population, patients with type 2 diabetes require access to anti-diabetes medications, yet they may be dissuaded from obtaining necessary prescriptions in harsh policy environments. Other studies of non-diabetic patients have documented delaying care until absolutely necessary, even for dire care needs, because of legal vulnerability (ie, documentation status).^15,37,38^ Additionally, few studies have examined differences in policies' effects on health, healthcare access, or diabetes care, by race/ethnicity, despite the potential for racism and the anti-immigrant climate to affect healthcare-seeking simultaneously.^39^

To address these gaps, this retrospective cohort study compared type 2 diabetes medication prescription trends between 2017-2019 in different county-level, policing-related sanctuary policy environments among a sample of patients with type 2 diabetes seen at clinics within OCHIN, a multi-state network of community health centers (CHC). Within OCHIN CHCs, there is a high prevalence of chronic diabetes complications (73%) among diabetic patients, underscoring the importance of access to appropriate pharmaceutical treatment.^40^ Previous studies within OCHIN CHCs have shown that Latine patients receive prescriptions for metformin at rates equal to or higher than non-Latine Whites and receive prescriptions as promptly as non-Latine Whites, but differences by local policy environment, nativity status, and other races/ethnicities have not been examined.^11^ Our study period (2017-2019) was marked by a heightened federal focus on immigration enforcement, thus we hypothesized that patients with type 2 diabetes who lived in the counties with more policing-related sanctuary policies had higher odds of receiving a prescription for anti-diabetes medications than patients living in counties with fewer sanctuary policies. We further examined whether this association varied by patient's nativity and race/ethnicity.

## Methods

### Study design and setting

This retrospective cohort study followed patients with type 2 diabetes seen at OCHIN clinics between January 2017 and December 2019. OCHIN is a multi-state network of CHCs that serves more than 6 million patients nationwide and provides a single patient ID number and shared medical records for each patient across all clinics.^41,42^ Community health centers, including OCHIN-affiliated CHCs, provide primary care to millions of vulnerable, uninsured, and underserved patients, including immigrants.^41-48^ The study period started immediately following the high adoption of county-level sanctuary policies in the mid-2010s, which occurred in response to restrictive federal immigration enforcement programs.^27^ The study period ended just prior to the peak rates of COVID-19 in the United States, which impacted care-seeking for many patients.^49^

### Study population

The cohort was limited to patients who had received a type 2 diabetes diagnosis. We used the International Classification of Diseases, Ninth Revision, Clinical Modification (ICD-9-CM) and the International Classification of Diseases, Tenth Revision, Clinical Modification (ICD-10-CM) diagnostic codes to identify type 2 diabetes patients diagnosed before the end of the study period in OCHIN electronic health records (EHR).^50^ The cohort included patients aged 18 and older who had their first encounter at OCHIN clinics before October 2019 to ensure sufficient follow-up time. Patients with missing nativity status (ie, country of birth) and missing address information in the EHR were excluded from the sample. To avoid exposure misclassification, we also excluded patients whose EHR-recorded county of residence changed during the study period. Additionally, we excluded patients who were pregnant at any point during the study period. We conducted a complete case analysis, for which we excluded patients with missing data on any covariate included in the fully adjusted models (Appendix A1).

### Variables

The study used county-level policy data as of December 2017 from the Immigrant Legal Resource Center (ILRC), a national nonprofit resource center tracking immigration enforcement policies at the county and city levels.^19^ The ILRC identifies the following seven policing-related sanctuary policies to assess county-level non-cooperation with ICE in jails: (1) no 287(g) agreements; (2) no ICE detention contracts; (3) limiting ICE detainers (ICE holds); (4) restricting notifications to ICE about release dates; (5) limiting ICE access to local jails and ICE interrogations of detainees; (6) prohibitions on any inquiries into immigration status and/or place of birth; (7) general prohibitions on participating in immigration enforcement.^19^ Policy start dates were not available in the dataset. We assume that the policy status of a county in 2017 serves as a reliable measure of the policy environment during the 2017-2019 study period. This assumption is supported by the fact that major changes in sanctuary policy enactments, particularly policing policies, occurred primarily in the mid-2010s and were relatively stable after 2015.^27^ Policy data were linked to patients' EHR using patients' county of residence.

We defined the policy exposure as a county-level sanctuary policy score (0-7). All policies were given the same weight because, to our knowledge, no published research has compared the impact of each of these policies in relation to one another. We also examined the exposure as a binary variable, defined as low (<3 policies) and high (3+ policies) sanctuary policy environments. The binary cutoff was chosen based on the distribution of policies across all US counties (not just those in our analytic sample).^19^ We could not make comparisons to having no sanctuary policies because of the small sample sizes of counties with no policies (see Appendix A2).

The outcome was a binary variable indicating whether a patient was prescribed a type 2 diabetes medication (ie, insulin or OHAs of any dose) at least once each year of the study period (annual prescriptions). We used prescription records in the EHR as a proxy for access to anti-diabetes medication because patients would have to engage with a provider to receive a prescription. Because patients may obtain refills at different cadences according to insurance coverage, we used a conservative minimum of receiving at least one prescription per year to manage blood sugar. The list of medications for type 2 diabetes was compiled according to the American Diabetes Association's recommended medications list and diabetes medication manufacturer websites (Appendix A3).

We included several patient- and county-level covariates, which we decided a priori were confounders. Patient-level covariates were age at first study encounter, sex (male/female), new/existing diabetes patient (diagnosis recorded during the study period vs before the study period or no date listed in the EHR), healthcare visits per year (<1, 1-3, 4-5, >5), the need for interpreter services (yes/no), insurance type (Medicaid, Medicare, other public insurance, private insurance, uninsured), total number of cardiovascular disease comorbidities, and measured hemoglobin A1C (HbA1c) more than 9% ever recorded in the patient's EHR between 2012-2019.^51^ Patients' US nativity status (non-US-born/US-born) and race/ethnicity (non-Latine Asian, non-Latine Black, Latine, non-Latine White) were also examined as effect-measure modifiers. County-level covariates of 5-year estimates (2014-2018) of the shares of the non-White population and the unemployment rate were included as measures of population demographics and socioeconomic conditions that might be common causes of enacting sanctuary policies in jails and prescriptions for anti-diabetes medications, thus introducing confounding. The 2017 age-adjusted diagnosed diabetes prevalence for adults was also included as a measure of county-level diabetes burden that could have influenced patient and provider prescription behaviors (Appendix A4).^52^

### Statistical analyses

We conducted multilevel logistic regression analyses to estimate associations between the county-level policing-related sanctuary policy environment and patients' receipt of diabetes prescriptions annually. We included clinic and patient-level random effects to account for within-cluster correlations of subject outcomes.^53,54^ First, we estimated crude odds ratios (ORs) for the sanctuary policy environment. Odds ratios were then adjusted for patient- and county-level covariates. Given the potential effects of racism on healthcare-seeking and provider-patient interactions and that immigrants of color may be impacted by both racism and sanctuary policies, we first checked for effect-measure modification separately by nativity status and race/ethnicity in fully adjusted models by adding an interaction term.^39,55^ Then, we tested for joint effects of sanctuary policies, nativity status, and race/ethnicity in fully adjusted models with three-way interactions. We additionally explored prescription trends by nativity and race/ethnicity in high and low-sanctuary policy environments using race/ethnicity-stratified analyses for non-Latine Black, Latine, and non-Latine White patients, as well as Latine Whites, Latine Blacks, and Other Latine groups. Non-Latine and Latine Asian patients were excluded from the three-way interaction model and race/ethnicity-stratified analyses because there were <10 type 2 diabetes cases in some nativity strata, resulting in imprecise estimates (Appendix A2).

We conducted sensitivity analyses by redefining the outcome to estimate the odds of ever getting prescribed type 2 diabetes medication during the entire 3-year study period. Here, we aimed to capture the patients who may have felt safe enough in their policy environment to go to a clinic at least once during the study period, even if they may not have felt safe enough to pursue regular care.

For all models, statistical significance was assessed at *P* ≤ .05, and 95% confidence intervals (CI) were reported to provide information on the margins of error. The data were analyzed using R version 4.2.1. The Northeastern University Institutional Review Board approved the study.

## Results

**Figure 1.**
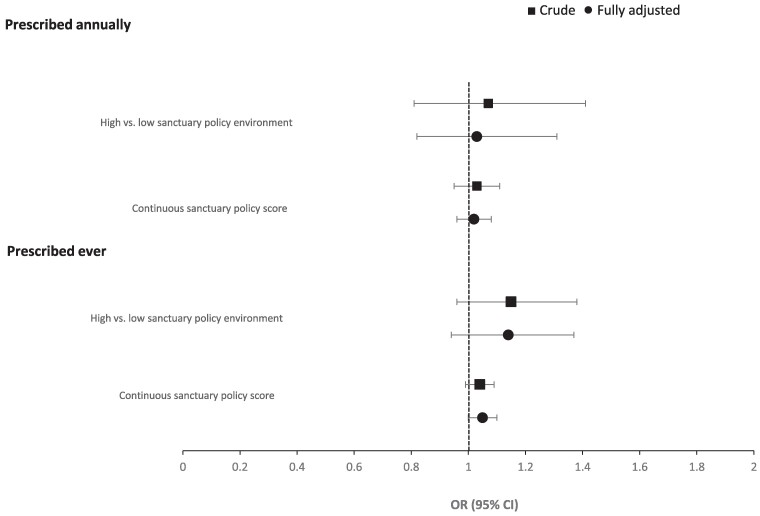
Associations between the county's sanctuary policy environment and anti-diabetes prescriptions for patients seen at OCHIN clinics (2017-2019). Source: Study-generated data. Estimates are adjusted for patient covariates (age, sex, US nativity status, race/ethnicity, type 2 diabetes diagnoses between 2017-2019, health center visits per year, the need for interpreter services, insurance type, cardiovascular disease comorbidities, HbA1c more than 9%), and county-level covariates (share of the non-White population, unemployment rate, age-adjusted diagnosed diabetes prevalence for adults).

Table 1 presents summary statistics for 25 189 type 2 diabetes patients seen at 363 OCHIN clinics from 2017 through 2019. The share of immigrant patients in the EHR was 61.0%. Most of the study population (84.1%) had Medicare, Medicaid, or other public insurance, and 7.3% were uninsured. There were 16 462 patients (65.4%) prescribed insulin or OHAs at least once during the entire study period. Each year, the share of patients receiving at least one prescription ranged from 45.4% to 46.7%. There were 91 counties in the dataset classified as high sanctuary and 60 classified as low-sanctuary environments (Appendix A4). Most patients (89.8%) lived in high-sanctuary policy counties, and slightly more than half lived in counties with all seven sanctuary policies in place (Table 1 and Appendix A2). Crude and fully adjusted ORs of getting type 2 diabetes prescriptions are presented in Figure 1. In the fully adjusted model, the OR for OCHIN clinic patients receiving a prescription annually with each additional county-level sanctuary policy was 1.04 (95% CI: 0.97-1.11). The OR for prescriptions in high- vs low-sanctuary policy environments was 1.14 (95% CI: 0.89-1.47). In models of patients receiving a diabetes prescription ever during the study period, each additional county-level sanctuary policy was again associated with slightly higher, but not statistically significant odds for receiving a prescription (OR: 1.05 [95% CI: 0.99-1.10]). Similar findings were observed in high- vs low-sanctuary environments (OR: 1.17 [95% CI: 0.96-1.43]).

**Table 1.** Characteristics of type 2 diabetes patients seen at OCHIN community health centers in 2017-2019, by US-nativity status.

Exhibit 1. Characteristics of type 2 diabetes patients seen at OCHIN community health centers in 2017-2019, by US-nativity status.			
	Total sample	US-born	Non-US-born
	*n* = 25 189	*n* = 9830	*n* = 15 359
**Demographic characteristics**			
Male (*n* (%))	11 351 (45.1)	4838 (49.2)	6513 (42.4)
Age at first study encounter:			
18-29	622 (2.5)	424 (4.3)	198 (1.3)
30-39	2095 (8.3)	959 (9.8)	1136 (7.4)
40-49	5347 (21.2)	2145 (21.8)	3202 (20.8)
50-59	8366 (33.2)	3674 (37.4)	4692 (30.5)
60-69	6337 (25.2)	2105 (21.4)	4232 (27.6)
>69	2422 (9.6)	523 (5.3)	1899 (12.4)
Race/ethnicity (*n* (%)):			
Non-Latine Asian	3370 (13.4)	240 (2.4)	3130 (20.4)
Non-Latine Black	5833 (23.2)	4023 (40.9)	1810 (11.8)
Latine	12 275 (48.7)	2545 (25.9)	9730 (63.4)
Non-Latine White	3711 (14.7)	3022 (30.7)	689 (4.5)
**Health Indicators**			
Type 2 diabetes diagnoses between 2017-2019 (*n* (%))	6490 (25.8)	2223 (22.6)	4267 (27.8)
^a,b^At least 1 comorbidities *n* (%)	23 185 (92.0)	2.42 (1.35)	2.12 (1.18)
^a^Hemoglobin A1c level more than 9% (*n* (%))	12 075 (47.9)	4790 (48.7)	7285 (47.4)
**Language and Healthcare Factors**			
Patients needing interpreter services (*n* (%))	1752 (7.0)	424 (4.3)	1328 (8.6)
^a^Insurance type			
Medicaid	11 882 (47.2)	5401 (54.9)	6481 (42.2)
Medicare	5565 (22.1)	2577 (26.2)	2988 (19.5)
Other public	3735 (14.8)	192 (2.0)	3543 (23.1)
Private	2180 (8.7)	971 (9.9)	1209 (7.9)
Uninsured	1827 (7.3)	689 (7.0)	1138 (7.4)
^a^Healthcare visits per year (*n* (%))			
<1	1495 (5.9)	692 (7.0)	803 (5.2)
1 to 3	6430 (25.2)	3012 (30.1)	3418 (22.1)
3.01 to 5	6418 (25.5)	2992 (30.4)	3426 (22.3)
>5	10 627 (42.2)	3693 (37.6)	6934 (45.1)
At least one anti-diabetes medication prescription received ever during the study period	16 462 (65.4)	5880 (59.8)	10 582 (68.9)
At least one anti-diabetes medication prescription received per year (*n* (%))			
2017	11 775 (46.7)	4605 (30.0)	7170 (72.9)
2018	11 438 (45.4)	4155 (27.1)	7283 (74.1)
2019	11 598 (46.0)	3200 (20.8)	8398 (85.4)
≥3 sanctuary policies in the county (n (%))	22 625 (89.8)	8390 (85.4)	14 235 (92.7)

Source: Study-generated data. Data are from 363 community health centers in the US. ^a^Anytime between 2012-2019. ^b^Heart disease, heart failure, cerebrovascular disease, peripheral vascular disease, hypertension, hyperlipidemia, kidney disease, obesity. Relevant groups of characteristics are in bold.

Models did not show any statistically significant effect-measure modification by nativity status on the association between sanctuary policies and receiving prescriptions annually or ever during the study period (Table 2). Sanctuary policies did not result in any meaningful increase in diabetes prescriptions for US- or non-US-born patients. Notably, nativity status was independently associated with receiving prescriptions. Non-US-born patients had higher odds of receiving a type 2 diabetes prescription annually than US-born patients in both high- (OR: 1.67 [95% CI: 1.48-1.88]) and low-sanctuary policy environments (OR: 1.60 [95% CI:1.16-2.19]). Estimates of ever receiving anti-diabetes prescriptions in the study period were similar (Table 2).

**Table 2.** Associations between counties' sanctuary policy environment, patients' US-nativity status, and prescriptions for anti-diabetes medications for patients with type 2 diabetes seen at OCHIN clinics (2017-2019).

	Model 1. Crude OR (95% CI)	Model 2. Adjusted for US-nativity status	Model 3. Fully adjusted for all covariates	Model 4. Fully adjusted and with an interaction term for US-nativity status and sanctuary policy environment				*P*-value for interaction term between sanctuary policy environment and patient's US-nativity status for Model 4
Prescribed annually				Non-US-born^a^	US-born^a^	High-sanctuary policy environment^a^	Low-sanctuary policy environment^a^	
Continuous sanctuary policy exposure	1.03 (0.95-1.12)	1.01 (0.93-1.10)	1.04 (0.97-1.11)	1.01 (0.91-1.12)	1.01 (0.95-1.08)	—	—	0.914
Non-US vs US-born	—	**2.05** (1.84-2.29)***	**1.63** (1.46-1.82)***	—	—	—	—	
High- vs low-sanctuary policy exposure	1.20 (0.89-1.61)	1.13 (0.85-1.52)	1.14 (0.89-1.47)	1.01 (0.69-1.50)	1.20 (0.91-1.58)	—	—	0.445
Non-US vs US-born	—	**2.05** (1.84-2.29)***	**1.63** (1.46-1.82)***	—	—	1.67 (1.48-1.88)***	1.60 (1.16-2.19)**	
ICC	0.71		0.6	0.63	0.54	0.59	0.71	0.6
**Prescribed ever**								
Continuous sanctuary policy exposure	1.03 (0.98-1.09)	1.02 (0.97-1.07)	**1.05 (0.99-1.10).**	1.02 (0.94-1.10)	1.03 (0.98-1.09)	—	—	0.930
Non-US vs US-born	—	**1.77** (1.64-1.92)***	**1.55** (1.41-1.71)***	—	—	—	—	
High- vs low-sanctuary policy exposure	**1.19 (0.98-1.44).**	1.13 (0.93-1.38)	1.17 (0.96-1.43)	1.13 (0.82-1.54)	1.18 (0.94-1.470	—	—	0.250
Non-US vs US-born	—	**1.77** (1.64-1.92)***	**1.55** (1.41-1.71)***	—	—	1.60 (1.44-1.79)***	1.39 (1.10-1.77)**	
ICC	0.15		0.11	0.14	0.63	0.1	0.22	0.11

Source: Study-generated data. *n* = 25 189. Full models are adjusted for patient covariates (age, sex, race/ethnicity, type 2 diabetes diagnoses between 2017-2019, health center visits per year, the need for interpreter services, insurance type, cardiovascular disease comorbidities, HbA1c more than 9%) and county-level covariates (share of the non-White population, unemployment rate, age-adjusted diagnosed diabetes prevalence for adults). Bold values indicate statistical significance. ICC scores represent intraclass correlation for clustering for each full and stratified sample, calculated separately for primary (annual) and sensitivity (ever) analyses. Significance codes: 0 “***” 0.001 “**” 0.01 “*” 0.05 “.” 0.1 “ ” 1. ^a^stratified estimates.

**Figure 2.**
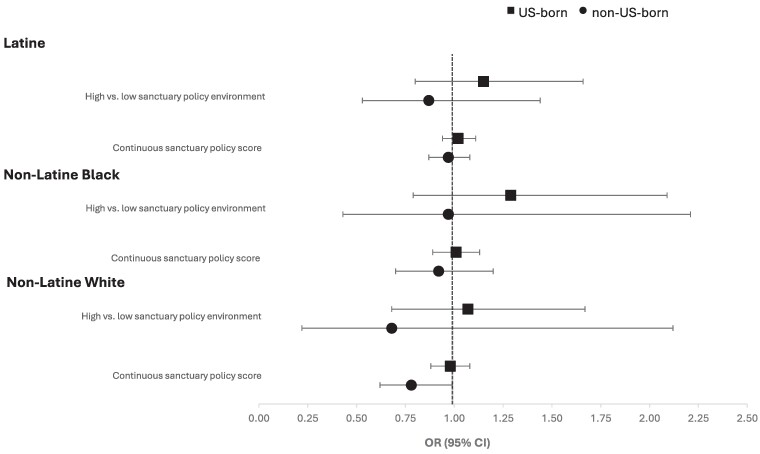
Fully adjusted, race/ethnicity-stratified associations between the county's sanctuary policy environment, patient's US nativity status, and annual anti-diabetes prescriptions for patients seen at OCHIN clinics (2017-2019). Source: Study-generated data. *n* (Latine) = 12 275. *n* (non-Latine Black) = 5833. *n* (non-Latine White) = 3711. Estimates are adjusted for patient covariates (age, sex, type 2 diabetes diagnoses between 2017-2019, health center visits per year, the need for interpreter services, insurance type, cardiovascular disease comorbidities, HbA1c more than 9%) and county-level covariates (share of the non-White population, unemployment rate, age-adjusted diagnosed diabetes prevalence for adults).

No statistically significant effect-measure modification was found by race/ethnicity in the fully adjusted models of sanctuary policies' association with anti-diabetes medication prescriptions, either annually or ever during the study period (Appendix A5). Testing for the three-way interactions of sanctuary policy, nativity, and race/ethnicity did not show statistically significant effect-measure modification in annual or ever-prescribed analyses (Appendix A6), but there were some differences observed when stratified by race/ethnicity (Appendix A7). Figure 2 visualizes the associations of sanctuary policies on the annual receipt of diabetes prescriptions, stratified by race/ethnicity and nativity. For all groups except for non-Latine White immigrants, we did not observe any statistically significant associations between sanctuary policies and prescription rates. Among non-Latine White immigrants, there were decreased odds of receiving a prescription annually with each additional sanctuary policy (OR: 0.78 [95% CI: 0.82-0.99]). With further stratification of Latines by race, Black and White Latines did not show any significant association of sanctuary environment by nativity status (Appendix A7). For immigrant Latines of “Other” races (excluding Asian), living in a high- vs low-sanctuary policy environment was associated with increased odds of receiving prescriptions annually (OR: 2.44 [95% CI:1.13-5.28]) (Appendix A7).

## Discussion

Sanctuary policies at the sub-federal level, adopted widely in the mid-2010s to limit cooperation with federal ICE, have been documented to reduce arrests, lower levels of worry, and improve mobility among immigrants.^25,28-30^ However, their impact on adult immigrants' health and access to necessary medication prescriptions, including those related to diabetes outcomes, remains underexplored. This retrospective cohort study examined the receipt of anti-diabetes prescriptions across different sanctuary policy environments among adult patients with type 2 diabetes within a multi-state network of CHCs. We found that policing-related sanctuary policies were not statistically significantly associated with receiving anti-diabetes prescriptions annually or at least once during the study period. The effect-measure modifications by nativity or race/ethnicity were not statistically significant.

The lack of an observed association between sanctuary policies and the receipt of diabetes prescriptions contradicts our initial hypothesis, which was based on findings among children or a general (not solely medically vulnerable) patient population.^21,25^ Findings from these previous studies may not be generalizable to a diabetic patient population. One possible explanation for these differences could be that, after a diabetes diagnosis, individuals tend to seek medical care regardless of the sanctuary policy environment due to the detrimental impact of diabetes on their quality of life. This may be especially pronounced in more severe cases of type 2 diabetes. As a result, the urgency to obtain diabetes medication might outweigh concerns about immigration enforcement. If this is the case, our findings in this diabetic sample would underestimate the effects of sanctuary policies in a less medically vulnerable population. In the only other known study of sanctuary policies and diabetes outcomes, undocumented Mexican immigrants achieved similar clinical outcomes as documented Mexican immigrants and US-born Mexican Americans, but this study was only conducted in sanctuary areas.^29^ As such, there has been no previous work examining whether diabetes-related outcomes, including prescription access, differ across sanctuary policy environments. Future research should make comparisons across different immigration-related policy environments. Further, researchers should continue exploring for whom sanctuary policies matter the most, including those with more severe diseases, those who are at risk for disease, and the general patient population.

We observed that immigrant patients had higher odds of receiving diabetes prescriptions regardless of the sanctuary policy environment, highlighting other potential influences on the receipt of prescriptions for these patients. Studies show that immigrant adults are more likely than non-immigrants to rely on CHCs because of the cost and opportunities to receive culturally and linguistically appropriate care.^56,57^ Consequently, CHCs may be accessible and trusted sources of care, especially for immigrants, potentially leading to a higher likelihood of receiving glucose-lowering medications in both high- and low-sanctuary policy environments.^43^ Thus, our findings are not generalizable to other healthcare settings (eg, academic medical centers, community hospitals, urgent care facilities).

Alternatively, higher prescription rates for immigrants may reflect differences in delayed detection, disease severity, or potential for disease management through diet and exercise. Prior research indicates that immigrants and racial/ethnic minorities are at higher risk for diabetes complications due to prolonged undetected disease.^12,13^ Our finding might reflect that US-born individuals with better overall healthcare access may detect type 2 diabetes at earlier stages and have the opportunity to manage their disease through diet and exercise, reducing the need for medication. However, this hypothesis could not be fully explored due to the lack of detailed information on the patient's diet and exercise history in the EHR.

Last, the effect of sanctuary policies on the receipt of prescriptions did not differ by race/ethnicity alone or by nativity status and race/ethnicity together in most groups. Only the non-Latine White immigrants were found to have decreased odds of receiving prescriptions annually in a county with each additional sanctuary policy. Previous studies of sanctuary policies have not examined differences by race/ethnicity, though the effects of racialized policing and immigration enforcement on healthcare-seeking behaviors, especially among Latine immigrants, are well documented, showing that fear of deportation often prevents them from seeking necessary healthcare.^58-61^ Because CHCs have been trusted sources of care for immigrants and different racial/ethnic groups, we may not observe an effect of policies on healthcare-seeking behaviors among these CHC patients.^61^ Further research is needed to explore whether unobserved differences exist in other healthcare settings.

This study is among the first to utilize multi-state EHR data to examine the relationship between county-level, policing-related sanctuary policies and type 2 diabetes medication prescriptions. The OCHIN dataset offers advantages over national survey datasets, Medicaid claims data, or smaller local studies. It is non-self-reported longitudinal data that minimizes recall bias and allows for follow-up of a medically vulnerable sample over time. Analyzing over 25 000 patients from nearly 400 clinics provided a diverse study population across differing county-level, immigration-related policy environments. Unlike previous studies examining immigrant health and healthcare experiences in the United States of single racial/ethnic groups (mostly Latine), EHR data from OCHIN enabled us to look at anti-diabetes prescription trends for different racial/ethnic groups. Additionally, our sample included patients from both uninsured and publicly insured populations, potentially capturing individuals with unauthorized documentation status among the uninsured. Even with the advantages of linking policy data to EHR data, EHR data inherently exclude individuals who do not engage with the healthcare system, thus our findings are not generalizable to populations outside of clinical care. Instead, this analysis focuses on patients with a known diabetes diagnosis for whom continued receipt of anti-diabetes prescriptions would be expected as part of disease management. The study has three notable limitations. First, there remains the potential for unmeasured confounding to bias findings. We adjusted for various county- and patient-level confounders and included clinic and patient-level random effects in models to mitigate some of the unmeasured variability. Still, other factors like patients' immigration documentation status or other institutional or governmental policies and practices may still introduce confounding that we could not address. Second, examining the aggregate sanctuary policy score, measured at one-time point, does not isolate the effects of any specific sanctuary policy. Future research could explore patterns of policy implementation, co-adoption and co-occurrence of policies, and potential joint effects of two or more policies. Individual policy analyses should complement, not replace studies of cumulative policy environments. Future studies, with implementation dates, could compare trends before and after policy changes to better understand the causal effects of sanctuary policies on diabetes treatment. To achieve this, more work is needed from scholars across disciplines to update existing measures, disaggregate multilevel aggregate measures, and make these verified databases easily accessible to researchers.^62,63^ The third limitation is the lack of country-of-origin data for many patients in the EHR and the inability to distinguish between documented and undocumented immigrants, whose healthcare experiences may differ substantially.^36^ However, previously observed effects of high immigration enforcement environments potentially apply to both documented and undocumented immigrant patients because of racialized policing practices and other spillover effects in mixed-status families.^21,64^ Therefore, results estimated by examining the information for this large multi-state sample provide a lower bound for effects on undocumented immigrants if they are at least as responsive to their counties' sanctuary policy environment as documented immigrants.

## Conclusion

Sub-federal-level sanctuary policies have been documented to reduce arrests, lower levels of worry, and improve mobility among immigrants. However, their impact on immigrant health remains underexplored. In this retrospective cohort study of patients receiving care in community health centers, we did not find statistically significant associations between sanctuary policies and annual anti-diabetes medication prescriptions. Notably, immigrant patients had higher odds of receiving diabetes prescriptions regardless of the sanctuary policy environment, suggesting other potential influences on the receipt of prescriptions in these settings.

## Supplementary Material

qxae178_Supplementary_Data
