# Supplementary material for: Sanctuary policies and type 2 diabetes medication prescription trends among community health center patients
Source: Health Aff Sch. 2025 Jan 21;3(1):qxae178. doi: 10.1093/haschl/qxae178 (PMC11747669; doi:10.1093/haschl/qxae178)
Supplement: qxae178_Supplementary_Data [file qxae178_supplementary_data.zip › Revised Appendix Materials.docx]

# **Appendices**

## **Appendix A1**. The restriction criteria to obtain the main analytic sample.

##

**Appendix A2. Distribution of the counties and patients in the main analytic sample by sanctuary policy environment, US-nativity status, and race/ethnicity (2017-2019)**

SOURCE Study-generated data. NOTES Data are from 363 community health centers across 151 counties in the US.

## **Appendix A3**. Type 2 Diabetes Medications

## **Appendix A4**. County-level characteristics in different county-level sanctuary policy environments (2014-2018).

**Source** Study generated data from 151 counties across the US. Source for Diabetes Prevalence: The Centers for Disease Control and Prevention. Diagnosed Diabetes - Total, Adults Aged 20+ Years, Age-Adjusted Percentage, Natural Breaks, All Counties. 2017 [cited 2023 Oct 20]. Available from: https://gis.cdc.gov/grasp/diabetes/diabetesatlas-surveillance.html#

**Appendix A5**. Associations between the sanctuary policy environment, patient’s race/ethnicity, and prescriptions for anti-diabetes medications for patients seen at OCHIN clinics (2017-2019).

|  | **Model 1. Crude OR (95% CI)** | **Model 2. Adjusted for Race/ethnicity** | **Model 3. Fully adjusted for all covariates** | **Model 4. Fully adjusted and with an interaction term for race/ethnicity and sanctuary policy environment** | | | | | **P-value for interaction term between sanctuary policy environment and patient's race/ethnicity for Model 4** |
| --- | --- | --- | --- | --- | --- | --- | --- | --- | --- |
| **Prescribed annually** |  |  |  | **Non-Latine Black^** | **Latine^** | **Non-Latine White^** | **High sanctuary policy environment^** | **Low sanctuary policy environment^** |  |
| *Continuous sanctuary policy exposure* | 1.03 (0.95 – 1.12) | 1.01 (0.93 – 1.09) | 1.04 (0.97 – 1.11) | 1.03 (0.92 – 1.17) | 1.02 (0.93 – 1.11) | 0.97 (0.88 – 1.07) | - | - |  |
| *Asian vs. White* | - | **1.59 (1.30 – 1.94)***** | **1.41 (1.17 – 1.70)***** | - | - | - | - | - | 0.824 |
| *Black vs. White* | - | 1.13 (0.97 – 1.31) | 1.01 (0.88 – 1.16) | - | - | - | - | - | 0.364 |
| *Latine vs. White* | - | **1.92 (1.68 – 2.20)***** | **1.37 (1.20 – 1.56)***** | - | - | - | - | - | 0.252 |
| *High vs. low sanctuary policy exposure* | 1.20 (0.89 – 1.61) | 1.14 (0.85 – 1.53) | 1.14 (0.89 – 1.47) | 1.31 (0.85 – 2.01) | 1.07 (0.74 – 1.55) | 1.05 (0.68 – 1.63) | - | - |  |
| *Asian vs. White* | - | **1.59 (1.30 – 1.93)***** | **1.41 (1.17 – 1.70)***** | - | - | - | **1.43 (1.18 – 1.73)***** | 1.08 (0.47 – 2.50) | 0.318 |
| *Black vs. White* | - | 1.13 (0.97 – 1.31) | 1.01 (0.88 – 1.16) | - | - | - | 1.05 (0.90 – 1.21) | 0.82 (0.53 – 1.28) | 0.156 |
| *Latine vs. White* | - | **1.92 (1.68 – 2.19)***** | **1.37 (1.20 – 1.56)***** | - | - | - | **1.37 (1.19 – 1.57)***** | 1.32 (0.81 – 2.14) | 0.355 |
| **Prescribed ever** |  |  |  |  |  |  |  |  |  |
| *Continuous sanctuary policy exposure* | 1.03 (0.98 – 1.09) | 1.01 (0.96 – 1.06) | 1.05 (0.99 – 1.10). | 1.05 (0.97 – 1.14) | 1.04 (0.96 – 1.13) | 1.00 (0.94 – 1.07) | - | - |  |
| *Asian vs. White* | - | **1.47 (1.27 – 1.70)***** | **1.37 (1.16 – 1.62)***** | - | - | - | - | - | 0.134 |
| *Black vs. White* | - | **1.13 (1.02 – 1.25)*** | 1.06 (0.94 – 1.19) | - | - | - | - | - | 0.592 |
| *Latine vs. White* | - | **1.83 (1.66 – 2.01)***** | **1.46 (1.30 – 1.64)***** | - | - | - | - | - | 0.272 |
| *High vs. low sanctuary policy exposure* | 1.19 (0.98 – 1.44) | 1.14 (0.94 – 1.38) | 1.17 (0.96 – 1.43) | 1.23 (0.92 – 1.65) | 1.25 (0.87 – 1.79) | 1.12 (0.83 – 1.52) | - | - |  |
| *Asian vs. White* | - | **1.47 (1.27 – 1.69)***** | **1.37 (1.16 – 1.62)***** | - | - | - | **1.36 (1.14 – 1.62)**** | 1.44 (0.74 – 2.78) | 0.786 |
| *Black vs. White* | - | **1.13 (1.02 – 1.25)*** | 1.05 (0.93 – 1.18) | - | - | - | 1.06 (0.93 – 1.20) | 1.00 (0.73 – 1.37) | 0.411 |
| *Latine vs. White* | - | **1.82 (1.66 – 2.01)***** | **1.46 (1.30 – 1.64)***** | - | - | - | **1.47 (1.30 – 1.66)***** | 1.38 (0.97 – 1.96). | 0.177 |

**SOURCE** Study-generated data. **NOTES** n = 25,189. Full models are adjusted for patient covariates (age, sex, race/ethnicity, type 2 diabetes diagnoses between 2017-2019, health center visits per year, the need for interpreter services, insurance type, cardiovascular disease comorbidities, HbA1c more than 9%) and county-level covariates (share of the non-White population, unemployment rate, age-adjusted diagnosed diabetes prevalence for adults). Significance codes: 0 ‘***’ 0.001 ‘**’ 0.01 ‘*’ 0.05 ‘.’ 0.1 ‘ ’ 1. ^ stratified estimates.

## **Appendix A6.** Three-way interaction term estimates from the fully adjusted models for associations between counties’ sanctuary policies, US-nativity status, and race/ethnicity for patients seen at OCHIN (2017-2019).

SOURCE Study-generated data. NOTES n = 21,819. Full models are adjusted for patient covariates (age, sex, race/ethnicity, type 2 diabetes diagnoses between 2017-2019, health center visits per year, the need for interpreter services, insurance type, cardiovascular disease comorbidities, HbA1c more than 9%), county-level covariates (share of the non-White population, unemployment rate, age-adjusted diagnosed diabetes prevalence for adults) and additionally adjusted for the interaction term between sanctuary policy status, nativity and race/ethnicity. Significance codes: 0 ‘***’ 0.001 ‘**’ 0.01 ‘*’ 0.05 ‘.’ 0.1 ‘ ’ 1. ^ stratified estimates.

**Appendix A7. Fully adjusted, race/ethnicity-stratified associations between the county’s sanctuary policy environment, patient’s US nativity status, and annual anti-diabetes prescriptions for patients seen at OCHIN clinics (2017-2019).**

**
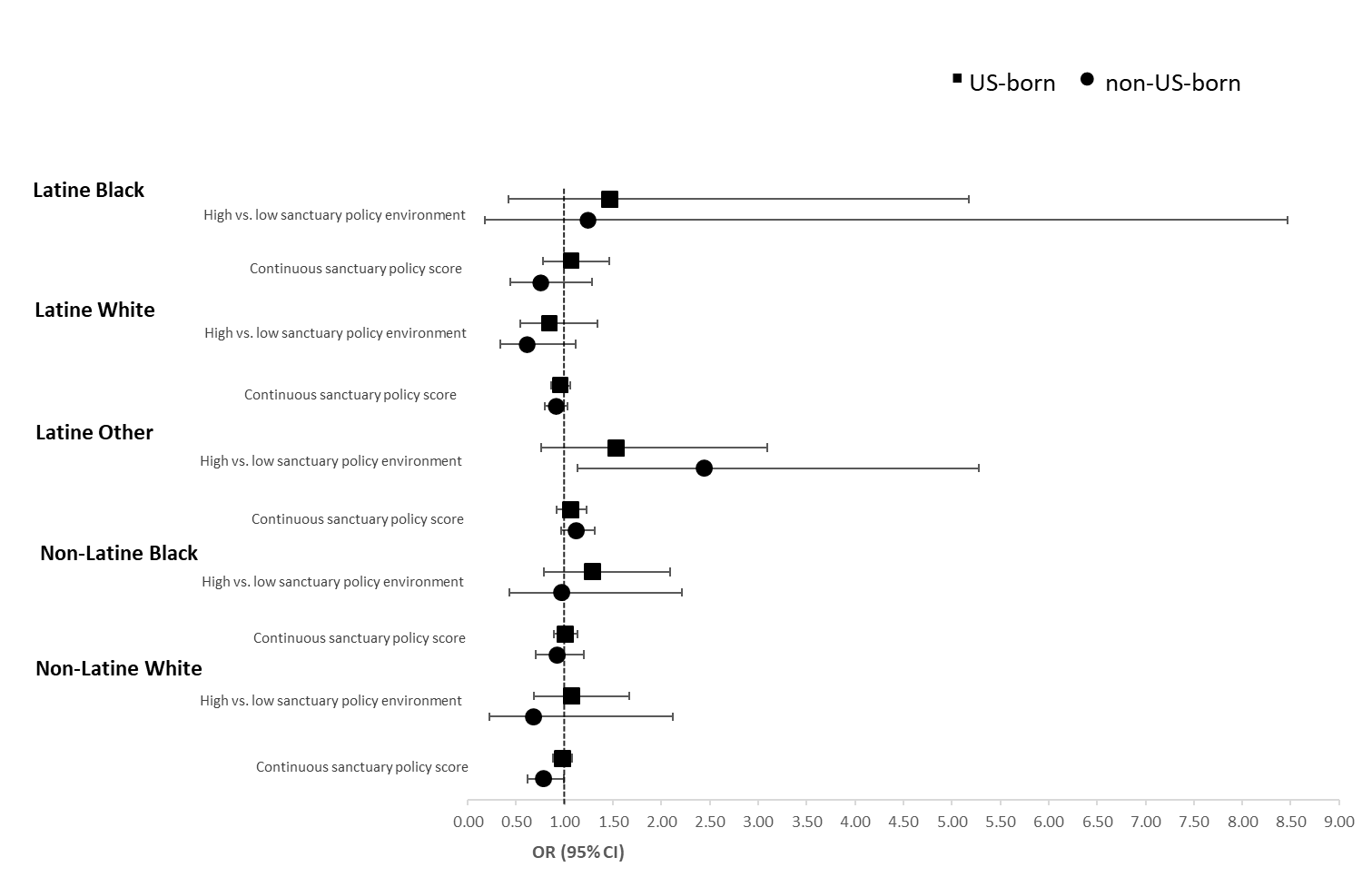
**

SOURCE Study-generated data. NOTES n (Latine Black) = 712. n (Latine White) = 10,230. n (Latine Other) = 1,311. n (non-Latine Black) = 5,833. n (non-Latine White) = 3,711. Latine and non-Latine Asians were excluded due to the small sample size. Estimates are adjusted for patient covariates (age, sex, type 2 diabetes diagnoses between 2017 - 2019, health center visits per year, the need for interpreter services, insurance type, cardiovascular disease comorbidities, HbA1c more than 9%) and county-level covariates (share of the non-White population, unemployment rate, age-adjusted diagnosed diabetes prevalence for adults).
